# SM22α Deletion Contributes to Neurocognitive Impairment in Mice through Modulating Vascular Smooth Muscle Cell Phenotypes

**DOI:** 10.3390/ijms24087117

**Published:** 2023-04-12

**Authors:** Xin Xu, Xiao-Qin Liu, Xin-Long Liu, Xu Wang, Wen-Di Zhang, Xiao-Fu Huang, Fang-Yue Jia, Peng Kong, Mei Han

**Affiliations:** 1 Department of Biochemistry and Molecular Biology, College of Basic Medicine, Hebei Medical University, Shijiazhuang 050017, China; 2 Key Laboratory of Neural and Vascular Biology of Ministry of Education, Shijiazhuang 050017, China; 3 Key Laboratory of Medical Biotechnology of Hebei Province, Hebei Medical University, Shijiazhuang 050017, China

**Keywords:** cognitive impairment, vascular smooth muscle cells, inflammation, smooth muscle 22-alpha, SRY-related HMG-box gene 10

## Abstract

Considerable evidence now indicates that cognitive impairment is primarily a vascular disorder. The depletion of smooth muscle 22 alpha (SM22α) contributes to vascular smooth muscle cells (VSMCs) switching from contractile to synthetic and proinflammatory phenotypes in the context of inflammation. However, the role of VSMCs in the pathogenesis of cognitive impairment remains undetermined. Herein, we showed a possible link between VSMC phenotypic switching and neurodegenerative diseases via the integration of multi-omics data. SM22α knockout (*Sm22α*^−/−^) mice exhibited obvious cognitive impairment and cerebral pathological changes, which were visibly ameliorated by the administration of AAV-SM22α. Finally, we confirmed that SM22α disruption promotes the expression of SRY-related HMG-box gene 10 (Sox10) in VSMCs, thereby aggravating the systemic vascular inflammatory response and ultimately leading to cognitive impairment in the brain. Therefore, this study supports the idea of VSMCs and SM22α as promising therapeutic targets in cognitive impairment to improve memory and cognitive decline.

## 1. Introduction

Cognitive impairment generally refers to various degrees of compromised cognitive function due to diverse factors, ranging from mild cognitive impairment to dementia [1]. Across the world, around 50 million people suffer from dementia, and this number is predicted to triple by 2050, representing a tremendous economic and social burden [2]. Unfortunately, despite hundreds of research studies over recent decades, there is no cure in sight. Considerable evidence now indicates that diseases with cognitive impairment, such as Alzheimer’s disease (AD), which is primarily a vascular disorder that precedes neuronal dysfunction, could be prevented or delayed by targeting the vascular component [3,4,5,6].

Vascular smooth muscle cells (VSMCs), which are critical regulators in the maintenance of vascular integrality and normal function [7], can switch from contractile to synthetic and proinflammatory phenotypes in the context of inflammatory injury. Smooth muscle 22 alpha (SM22α), also known as Transgelin, as a differentiated VSMC marker [8], is involved in regulating actin filament assembly and cytoskeletal rearrangement and is required for maintaining the differentiated phenotype of VSMCs [9]. Disruption of SM22α represents the phenotypic switching of VSMCs from contractile to synthetic or proinflammatory, resulting in vascular inflammation and oxidative stress that is associated with neointimal formation and hypertension [10,11]. A recent study demonstrated that loss of the VSMC contractile phenotype correlates with Tau accumulation in brain arterioles and that the expression of SM22α protein is inversely correlated with CD68 and Tau expression [12]. We hypothesize that deficiency of SM22α contributes to neuroinflammation pathogenesis via inducing VSMC phenotypic switching.

In this study, we demonstrated that SM22α disruption promotes the expression of SRY-related HMG-box gene 10 (Sox10) in VSMCs, thereby aggravating the systemic vascular inflammatory response and involving brain tissues, leading to cognitive impairment.

## 2. Results

### 2.1. Multi-Omics Points to a Possible Link between VSMC Phenotypic Switching and Neurodegenerative Diseases

**Figure 1:**
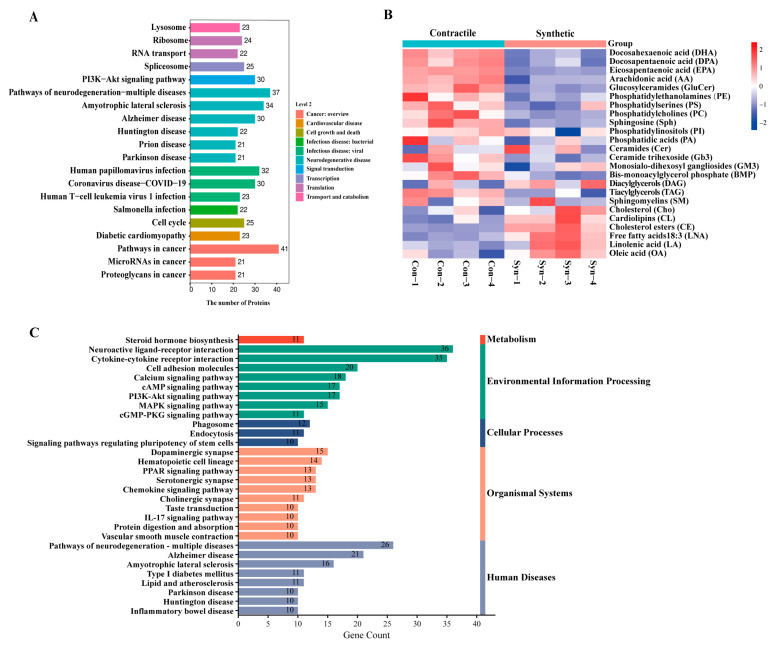
Multi-omics points to a possible link between VSMC phenotypic switching and neurodegenerative diseases. (**A**) KEGG pathway enrichment of differentially expressed proteins between contractile and synthetic VSMCs in mice from proteomic analyses (*n* = 3 independent samples). (**B**) Heatmap of each lipid class between contractile and synthetic VSMCs in mice from lipidomic analyses (*n* = 4 independent samples). (**C**) KEGG pathway enrichment of upregulated differentially expressed mRNAs in the arteries of *Sm22α*^−/−^ mice compared with WT mice from transcriptomic analyses (*n* = 8 mice per group).

To determine the association between the phenotypic switching of VSMCs and neuroinflammation, we first prepared the model of VSMCs switching from contractile to synthetic phenotypes in vitro, and generated the differential protein expression profiles of the contractile and synthetic VSMCs via proteomic analysis (Figure 1A). Kyoto Encyclopedia of Genes and Genomes (KEGG) pathway enrichment [13] showed that the phenotypic switching of VSMCs was associated with a variety of neurodegenerative diseases, including Huntington’s disease, Parkinson’s disease, and Alzheimer’s disease.

The brain is highly enriched in lipids, and imbalances in lipid homeostasis are related to neurodegenerative diseases. Previous studies have shown that the lipids represented by docosahexaenoic acid (DHA), phosphatidylethanolamine (PE), and phosphatidylcholine (PC) are generally considered to have neuroprotective effects and are reduced in the aging and diseased brain [14]. Next, we performed a lipidomic analysis of contractible and synthetic VSMCs (Figure 1B) and found a consistent conclusion that the beneficial DHA, PE, and PC were visibly decreased in the synthetic VSMCs with a significant accumulation of cholesterol esters (CE), contributing to the formation of foam cells in atherosclerosis [15].

We then took advantage of the transcriptomic analysis obtained from SM22α knockout (*Sm22α*^−/−^) mice compared with wild-type (WT) mice to directly evaluate the effects of SM22α. We found that the upregulated gene enrichment pathway in the aortas of the *Sm22α*^−/−^ mice focused on inflammatory and neurodegenerative diseases (Figure 1C and Supplementary Data S1), which further reinforced the idea that SM22α is an anti-inflammatory and vascular protective effector, pointing to a possible link between SM22α-related VSMC dysfunction and neurological impairment. 

### 2.2. Depletion of SM22α Leads to Pathological Changes in the Hippocampus Region of the Mouse Brain

**Figure 2:**
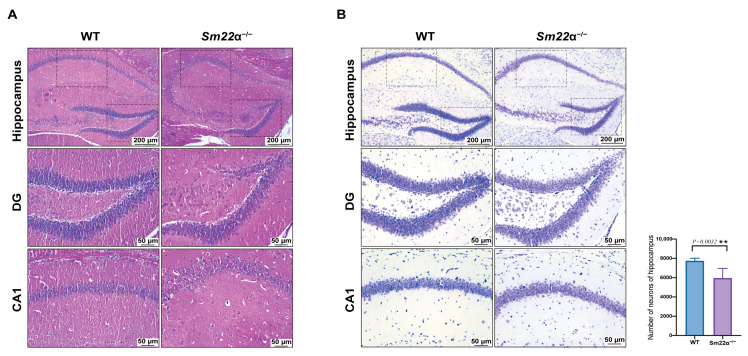
Depletion of SM22α leads to pathological changes in the hippocampus region of the mouse brain. (**A**) Representative images of HE staining in the hippocampus of the WT and *Sm22α*^−/−^ mice. (**B**) Representative images of Nissl staining in the hippocampus of the WT and *Sm22α*^−/−^ mice are shown on the left, with the statistical results regarding the number of neurons on the right. CA1, hippocampal CA1 layer; DG, dentate gyrus. ** *p* < 0.01.

To explore whether SM22α deficiency affects brain pathology, morphological analysis of the mouse hippocampus was performed using HE staining (Figure 2A) and Nissl staining (Figure 2B). The results showed that the number of neurons in the hippocampus of the *Sm22α*^−/−^ mice was significantly reduced, and the overall arrangement of neurons was not as tight as that in the WT group, especially at the edge, where it was meaningfully looser. However, we found no significant accumulation of amyloid-β (Aβ) peptide burden, the major neuropathological hallmark of AD, in the WT or *Sm22α*^−/−^ mice (Supplementary Figure S1).

### 2.3. Sm22α^−/−^ Mice Exhibit Cognitive Impairment

**Figure 3:**
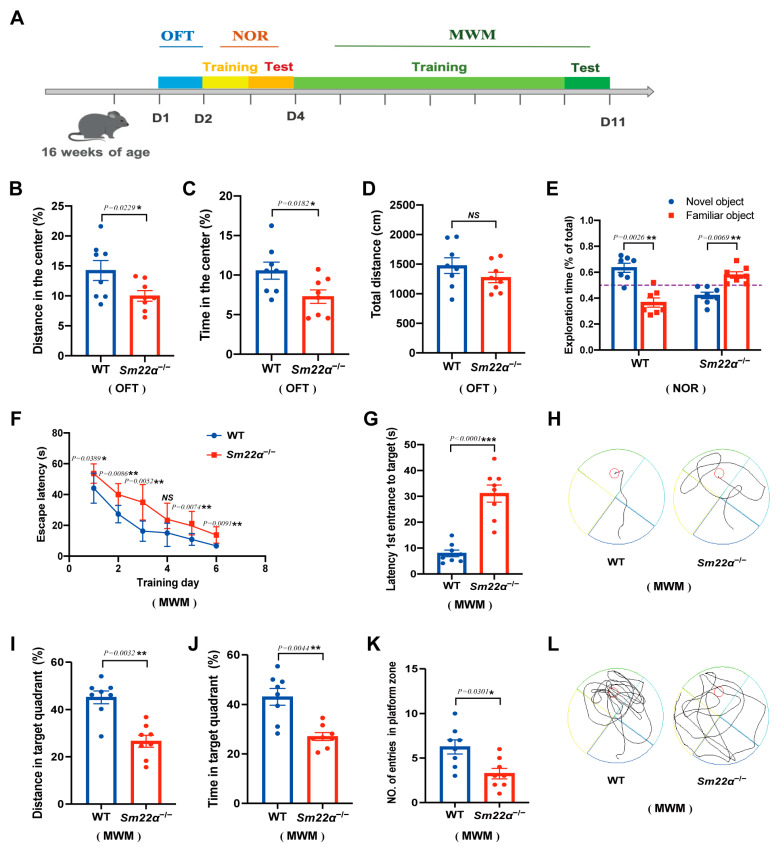
The *Sm22α*^−/−^ mice exhibited cognitive impairment. (**A**) Schematics of the experimental design for the cognitive behavioral study. OFT, open field test; NOR, novel object recognition test; MWN, Morris water maze test; D, day. (**B**–**D**) Performances in the OFT were recorded for 5 min. (**B**) Distance traveled in the center. (**C**) Time spent in the center. (**D**) Total travel distance. (**E**) Discrimination ratio of novel versus familiar objects during NOR. (**F**–**L**) Performances in the MWM during the training days and probe test. (**F**) Escape latency to the platform during the training days. (**G**) Latency to first reaching the platform and (**H**) representative track images of the mice in the probe test with the platform. (**I**) Distance in the target quadrant, (**J**) time spent in the target quadrant, (**K**) entry number in the platform zone, and (**L**) representative track images of the mice during the 60 s probe test without the platform. Data are represented as the mean ± SD (*n* = 8 mice per group). * *p* < 0.05, ** *p* < 0.01, and *** *p* < 0.001.

We then systematically evaluated the behavior of the *Sm22α*^−/−^ mice, including motor skills, sensory processing, and ability to perform various cognitive tasks, as shown in the experimental design schedule (Figure 3A). First, we assessed the locomotion activity and anxiety with an open field test (OFT). We found that the *Sm22α*^−/−^ mice preferred to avoid the center of an open area (Figure 3B,C), but observed no significant difference in the total travel distance compared to the WT mice (Figure 3D). It seems that SM22α deficiency causes mild anxiety but not impaired locomotor activity. As the mice developed habituation to familiar objects, they tended to explore more. In the novel object recognition test (NOR), the WT mice spent more time exploring novel objects than familiar ones. However, *Sm22α*^−/−^ mice spent significantly more time with the familiar object rather than exploring the novel object, which strongly supports a lack of recognition of novelty in *Sm22α*^−/−^ mice (Figure 3E). Finally, we examined the spatial learning and memory of the mice through the Morris water maze test (MWM). Compared to the WT mice, the *Sm22α*^−/−^ mice displayed obvious spatial learning and memory deficits, as reflected by their longer escape latency time during the training days (Figure 3F) and probe test (Figure 3G,H), less travel distance and time spent in the target quadrant (Figure 3I,J), and a lower entry number in the platform zone (Figure 3K,L) during the probe test without a platform. Together, these results indicate that the *Sm22α*^−/−^ mice exhibited cognitive impairment.

### 2.4. Administration of AAV-SM22α Ameliorates Cognitive Impairment in Sm22α^−/−^ Mice

**Figure 4:**
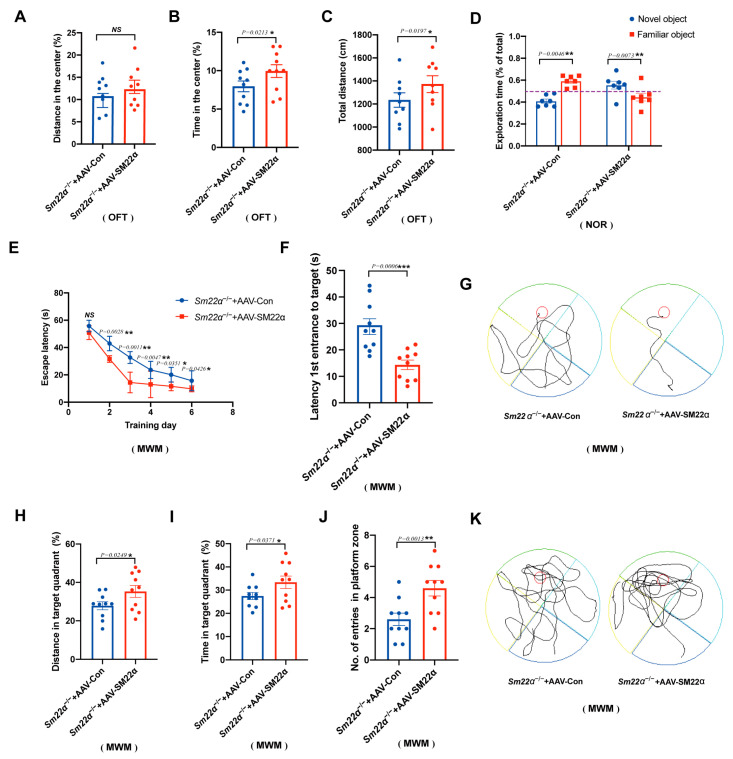
Administration of AAV-SM22α improved the cognitive impairment in the *Sm22α*^−/−^ mice. (**A**–**C**) Performance in the OFT was recorded for 5 min. (**A**) Distance traveled in the center. (**B**) Time spent in the center. (**C**) Total travel distance. (**D**) Discrimination ratio of novel versus familiar objects during the NOR. (**E**–**K**) Performance in the MWM in the training days and probe test. (**E**) Escape latency to the platform during the training days. (**F**) Latency to first reach the platform and (**G**) representative track images of the mice in the probe test with the platform. (**H**) Distance in the target quadrant, (**I**) time spent in the target quadrant, (**J**) entry number in the platform zone, and (**K**) representative track images of the mice during the 60 s probe test without the platform. Data are represented as the mean ± SD (*n* = 8 mice per group). * *p* < 0.05, ** *p* < 0.01, and *** *p* < 0.001.

To further identify a potential causative link between SM22α depletion and cognitive impairment, we selected an AAV carrying SM22α to perform a gain-of-function study in the *Sm22α*^−/−^ mice. As expected, the behavioral analyses showed that the administration of AAV-SM22α ameliorates cognitive impairment. After the *Sm22α*^−/−^ mice restored SM22α expression, the anxiety in the OFT test was mitigated (Figure 4A–C) and the exploration time of the novel object in the NOR test was extended (Figure 4D). The most obvious changes are in the spatial learning and memory of mice from the MWM test, as indicated by the shorter escape latency time during the training days (Figure 4E) and probe tests (Figure 4F,G), greater travel distance and higher amount of time spent in the target quadrant (Figure 4H,I), as well as a higher entry number in the platform zone (Figure 4J,K) in the probe test without a platform. Summing up, these behavioral tests demonstrate that SM22α protects against cognitive impairment in mice.

### 2.5. Sox10 Is Closely Associated with Neurodegenerative Diseases

**Figure 5:**
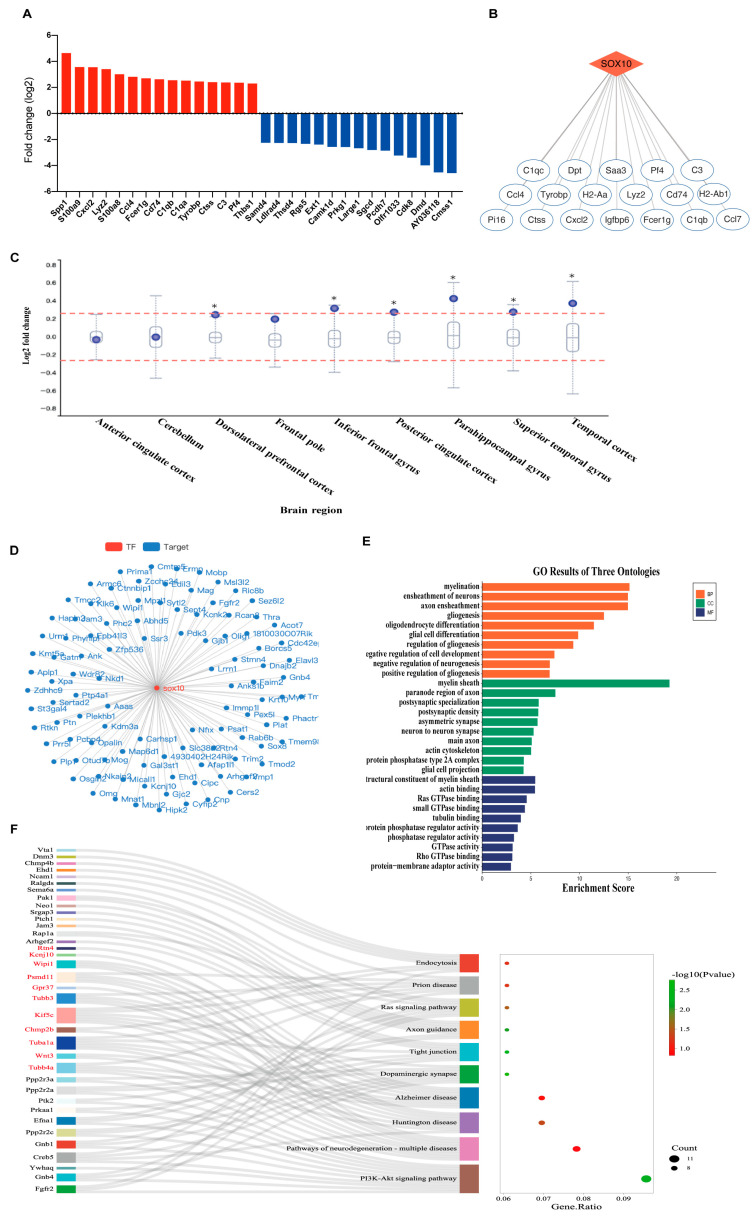
Sox10 is closely associated with neurodegenerative diseases. (**A**) Top differentially expressed genes in modulated VSMCs of the neointima of those mice with ligated carotid arteries from single-cell RNA sequencing (*n* = 30 mice). (**B**) Potential transcriptional regulatory target genes of Sox10 in modulated VSMCs. (**C**) Differential expression of Sox10 between AD cases and controls across brain regions from the AMP-AD knowledge portal. The box plot depicts how the differential expression of Sox10 (purple dot) compares to the expression of other genes in a given tissue. Meaningful differential expression is considered to be a log2 fold change value greater than 0.263 or less than –0.263 (red line). (**D**) The interaction network of transcription factor Sox10 and its downstream target genes detected in the mouse brain using the GRNdb online network (http://www.grndb.com/; accessed on 6 November 2022). (**E**) Gene ontology (GO) terms of the Sox10 downstream target genes in the mouse brain. BP, biological process; CC, cellular component; MF, molecular function. (**F**) Sankey plot of the pathways enriched for the Sox10 downstream target genes in the mouse brain. Genes associated with neurodegenerative diseases are shown in red.

Single-cell RNA sequencing of the neointima of those mice with ligated carotid arteries revealed that the differentially expressed genes (DEGs) were mainly immune inflammatory molecules in modulated VSMCs of the neointima, such as C1qc, C1qb, and Cxcl2 (Figure 5A and Supplementary Data S2), representing a proinflammatory VSMC phenotype. Furthermore, we predicted that transcription factor Sox10 would act as the strongest regulator to promote the expression of the immune inflammatory molecules in proinflammatory phenotypic VSMCs (Figure 5B). We then queried the publicly available AMP-AD Knowledge Portal to determine whether there was an association between Sox10 expression levels and AD (Figure 5C). We showed that the expression level of Sox10 in most brain regions of AD cases was significantly upregulated, compared to the controls. Next, we used the GRNdb [16] online network to identify 289 downstream target genes (Figure 5D) of Sox10 in the mouse brain. These target genes were subjected to gene ontology (GO) (Figure 5E) and pathway analyses (Figure 5F). Neurogenesis- and actin-related GO terms were significantly enriched, as shown by the negative regulation of neurogenesis-associated biological processes (BP), actin cytoskeleton-associated cellular components (CC), and actin binding-associated molecular functions (MF). Sankey plots showcased neurodegenerative disease-related signaling pathways that were remarkably enriched.

### 2.6. Sox10 Expression Is Associated with the Inflammatory Response in the Mouse Brain

**Figure 6:**
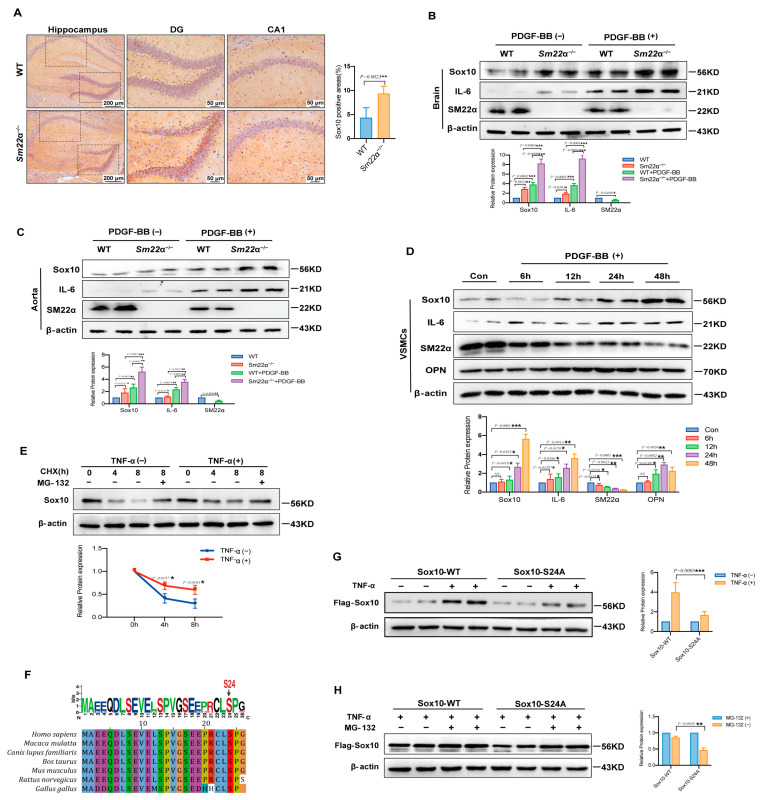
Sox10 expression is associated with the inflammatory response in the mouse brain. (**A**) Representative images of Sox10 immunohistochemical staining in the hippocampus of the WT and *Sm22α*^−/−^ mice are shown on the left, with the corresponding statistical result on the right. (**B**) Representative Western blots and quantitative analysis of the brain in the WT and *Sm22α*^−/−^ mice treated with PDGF-BB ex vivo. (**C**) Representative Western blots and quantitative analysis of the aorta in the WT and *Sm22α*^−/−^ mice treated with PDGF-BB ex vivo. (**D**) Representative Western blots and quantitative analysis of VSMCs treated with PDGF-BB at different time points. (**E**) Representative Western blots and quantitative analysis of Sox10 in HEK 293A cells treated with TNF-α after pretreatment with CHX or MG132 for the indicated times. (**F**) Sequence alignment of Sox10 Ser24 from different species. (**G**) Representative Western blots and quantitative analysis of Sox10 in HEK 293A cells transfected with Flag-Sox10-WT or Flag-Sox10-S24A, and then treated with TNF-α. (**H**) Representative Western blots and quantitative analysis of Sox10 in HEK 293A cells transfected with Flag-Sox10-WT or Flag-Sox10-S24A, and then pretreatment with or without MG132 before adding TNF-α. Data are represented as the mean ± SD. * *p* < 0.05, ** *p* < 0.01, and *** *p* < 0.001.

In view of the critical regulatory role of Sox10 in the proinflammatory phenotypic switching of VSMCs, and the association between Sox10 and brain inflammation in the clinical public database and bioinformatics analysis, we examined the expression of Sox10 in the brain of the *Sm22α*^−/−^ mice. It was observed that Sox10 expression was significantly increased in the hippocampus of the *Sm22α*^−/−^ mice compared to the WT mice (Figure 6A). Platelet-derived growth factor-BB (PDGF-BB) ex vivo-treated aortas and brains are successful models of dysfunctional VSMCs and neuroinflammation [12]. We showed that the expression of Sox10 and the proinflammatory cytokine interleukin-6 (IL-6) were strikingly elevated in the aortas and brains of the mice after 48 h of treatment with PDGF-BB (Figure 6B,C). Notably, the *Sm22α*^−/−^ mice exhibited a stronger intensity of Sox10 and IL-6 increasements in response to PDGF-BB stimulation (Figure 6B,C). In line with the results in the ex vivo models, Sox10 expression was significantly increased in VSMCs upon PDGF-BB treatment, along with the upregulation of the synthetic marker OPN and proinflammatory marker IL-6 and the downregulation of the contractility marker SM22α (Figure 6D), suggesting that Sox10 expression is associated with the inflammatory response in VSMCs and brain tissues. 

Sox10 is tightly regulated at the post-translational level. To determine whether protein stability contributes to increased Sox10 expression in the context of inflammatory stimulation, we evaluated the half-life of Sox10 in HEK 293A cells using cycloheximide (CHX) and proteasome inhibitor MG132. Sox10 was rapidly degraded by the ubiquitin-proteasome pathway in TNF-α-untreated cells, which was reversed by MG132, whereas TNF-α treatment enhanced the stability of Sox10 protein, as shown via the Western blotting of Sox10 protein (Figure 6E). A previous study predicted that S24 is an important phosphorylation site of Sox10 [17], which may affect its protein stability, and bioinformatics analysis also showed that S24 is highly conserved among species (Figure 6F). We then mutated the S24 site to mimic inactivation to further determine the relationship between S24 phosphorylation and TNF-α-enhanced Sox10 stability. The results showed that the increase in the Sox10-S24A mutant was lower than that of the wild-type protein (Sox10-WT) in MG132-treated cells upon stimulation of TNF-α in MG132-treated cells, implying increased protein degradation (Figure 6G,H). Collectively, these findings suggest that the stability of Sox10 protein is S24 phosphorylation-dependent in the context of inflammation.

## 3. Discussion

In the current study, integrated multi-omics uncovered that the phenotypic switching of VSMCs is associated with neurodegenerative diseases. We showed that the depletion of SM22α is a link between VSMC dysfunction and neurodegenerative diseases, with the *Sm22α*^−/−^ mice exhibiting cognitive impairment. Importantly, the administration of AAV-SM22α improved cognitive function in the *Sm22α*^−/−^ mice. Mechanistically, the Sox10 expression increased via reduced degradation of the protein in an S24 phosphorylation-dependent manner in the VSMCs of the *Sm22α*^−/−^ mice. Moreover, Sox10 mediated the phenotypic switching of the VSMCs, contributing to exaggerated inflammation both in the aorta and brain. 

Neurovascular units (NVUs) comprise vascular cells (including the endothelium, pericytes, and VSMCs), glial cells, and neurons, which closely communicate with one another [18]. Given their inherent phenotypic plasticity, VSMCs can switch their phenotypes in response to a variety of pathogenic stimuli and mediate tissue repair and inflammatory damage [19,20]. In this study, we indicated that modulated VSMCs are closely related to neurodegenerative disease, suggesting that VSMCs in NVUs may be one of the core drivers of neurovascular disease initiation and progression.

The phenotypic switching of VSMCs is characterized by loss of contractile markers such as SM22α. We and others have demonstrated that disruption of SM22α enhances the inflammatory response in VSMCs [21,22,23]. Conversely, overexpression of SM22α significantly contributes to alleviating neointimal formation and hypertension via inhibiting the proliferation, inflammation, and oxidative stress of VSMCs [15,24,25,26]. Our findings expand on previous work that *Sm22α*^−/−^ mice display a higher risk of not only cardiovascular diseases [15,27], but also neurodegenerative diseases. Through behavioral experiments and pathological examinations, we showed that SM22α has an independent effect on brain function. Although the *Sm22α*^−/−^ mice had a normal motor function, their cognitive abilities were significantly impaired. The pathological results showed that the neurons were damaged in the *Sm22α*^−/−^ mice. Nevertheless, whether SM22α disruption also underlies cognitive impairment progression remains to be further explored.

Sox10 is grouped with Sox8 and Sox9 in the SoxE class of the Sox family [28], which plays a critical role in nervous system development [29]. Sox10, as a neural crest lineage transcription factor, is involved in the development of neural crest (NC), as well as various NC derivatives [30], and the proliferation, survival, and differentiation of a wide variety of cells [31]. Sox10 is highly expressed in a variety of tumors and plays important roles in tumor initiation, maintenance, and progression to advanced stages of melanoma [32,33]. Various post-translational modifications, such as acetylation, phosphorylation, and sumoylation, modulate the activity, stability, and intracellular localization of Sox10, and the relevance of most has not been functionally assessed [30]. In addition, it has been pointed out that Sox10-positive cells are one of the sources of neointima cells after vascular injury [34,35]. However, the role of Sox10 in neurodegenerative disease has never been reported. Using single-cell RNA sequencing, we characterized the transcriptomic phenotype of VSMCs from the neointima of mice and demonstrated that the DEGs in modulated VSMCs were mainly proinflammatory factors. Furthermore, we showed that Sox10 acts as a top regulatory hub with the highest number of target genes involved in proinflammatory VSMCs. Multiple bioinformatics predictive analyses indicated that Sox10 is closely related to neurodegenerative diseases. We also showed that Sox10 is highly expressed in *Sm22α*^−/−^ aortas and brain tissues. All of these lines of evidence point to the key role of Sox10 in mediating the neurovascular inflammatory response.

Although there have been studies on the role of vascular dysfunction in cognitive impairment in the past [4,36], they were mostly limited to affecting the blood supply to the brain. Herein, we showed that the inflammation mediated by VSMCs has a significant influence on the progression of cognitive impairment. One limitation of this study is that only *Sm22α*^−/−^ mice aged 3–4 months were used instead of 18–24-month-old mice or dementia model mice, as normally designed. This may be one of the reasons why brain tissue lesions were not obvious. We hope further explorations can be carried out in follow-up research through strict and unified standards. 

## 4. Materials and Methods

### 4.1. Experimental Animals

*Sm22α*^−/−^ mice (B6.129S6-Taglntm2(cre)Yec/J), aged 12–14 weeks, with a Cre-recombinase gene inserted into the endogenous Transgelin (SM22α) locus, were purchased from the Jackson Laboratory [37]. Age-matched C57BL/6J WT mice were purchased from Charles River. The animals were housed in a pathogen-free environment with the ambient temperature maintained at 21–23 °C and the relative humidity at 50–60%, with a 12 h/12 h light/dark cycle. The animals were allowed ad libitum access to water and standard laboratory chow (16% protein, 4% fat, and 6% fiber), unless otherwise indicated. Mice aged 16–18 weeks were used for studies. All of the animal experiments were approved by the Ethics Committee for Animal Experiments and the Institutional Animal Care and Use Committee of Hebei Medical University and were carried out according to the guidelines of Directive 2010/63/EU of the European Parliament on the protection of animals used for scientific purposes.

### 4.2. Proteomic Analyses

VSMCs were cultured (shown below in Section 4.9) and stimulated with PDGF-BB (20 ng/mL, Sigma, Saint Louis, MO, USA) to induce a synthetic phenotype, shown as a decreased expression of contractile markers (SM22α and MYH11) and increased expression of synthetic markers (OPN and PCNA). Three independent samples were repeated for each group. Cells were collected and protein pellets were sonicated and digested into peptides as previously described [38]. The peptide mixture (100 μg) was labeled using a TMT reagent (Thermo Scientific, Waltham, MA, USA). Then, LC-MS/MS analysis was performed on a Q Exactive mass spectrometer (Thermo Scientific, Waltham, MA, USA) that was coupled to Easy nLC (Thermo Scientific, Waltham, MA, USA) for 60/90 min. The acquired LC-MS/MS analysis data were searched using the MASCOT engine (Matrix Science, UK; version 2.2) embedded into Proteome Discoverer 1.4 software (Thermo Scientific, Waltham, MA, USA; version 1.4) for identification and quantitation analysis. Then, the proteins were blasted against the online KEGG database (http://geneontology.org/; accessed on 5 April 2022) to retrieve their KEGG orthology identifications and were subsequently mapped to pathways in the KEGG. Enrichment analysis was applied based on Fisher’s exact test, considering all quantified proteins as the background dataset. Benjamini–Hochberg correction for multiple testing was further applied to adjust the derived *p*-values, and only pathways with *p*-values under a threshold of 0.05 were considered significant.

### 4.3. Lipidomic Analyses

The VSMCs were stimulated with PDGF-BB (20 ng/mL, Sigma, Saint Louis, MO, USA), the same as the proteomic analysis treatment. Four independent samples were repeated for each group. Cells were collected and lipids were extracted using a modified Bligh and Dyer extraction procedure (double rounds of extraction) [39,40]. Briefly, cells were incubated in 750 µL of chloroform/methanol 1:2 (*v*/*v*) with 10% deionized water for 30 min. At the end of the incubation, 350 µL of deionized water and 250 µL of chloroform were added. The samples were then centrifuged and the lower organic phase containing lipids was extracted into a clean tube. Lipid extraction was carried out twice, and the lipid extracts were pooled into a single tube and dried in the SpeedVac under OH mode. Then, polar lipids were analyzed using an Exion UPLC system coupled with a triple quadrupole/ion trap mass spectrometer (6500 Plus Qtrap; SCIEX, Framingham, MA, USA) as described previously [40]. Individual lipid species were quantified by referencing spiked internal standards. Heatmaps were produced using the R package pheatmap.

### 4.4. Transcriptomic Analyses

The aortas of the mice were collected from the *Sm22α*^−/−^ mice and their littermate wild-type controls (*n* = 8 mice per group, aged 12 weeks) as described previously [27]. Then, the adventitia and endothelium were quickly stripped and separated from the thoracic aortas in ice-cold RNase-free PBS. The harvested tissues were placed in RNAlater (Ambion, Austin, TX, USA) in preparation for RNA-seq analysis. RNA-seq libraries were sequenced onto an Illumina HiSeq2000 instrument and subjected to 100 cycles of paired-end (2 × 100 bp) sequencing. The processing of fluorescent images into sequences, base-calling, and quality value calculations were performed using the Illumina data processing pipeline (version 1.8).

### 4.5. Single-Cell RNA Sequencing

Carotid artery ligation is an acknowledged model of neointimal formation that is often used to examine phenotypic switching of VSMCs in vivo. C57BL/6J WT mice (*n* = 30 mice, aged 10–12 weeks) were anesthetized using 2.5–3% isoflurane via inhalation. The left common carotid artery was tied firmly with one knot using a 6-0 silk suture just below the bifurcation point. All animals were euthanized via isoflurane overdose on day 28 after ligation, and then we followed a published protocol, as previously described [41], to isolate cells from the neointima of mouse carotid arteries to prepare single-cell suspensions for Chromium Single Cell Controller (10× Genomics) single-cell RNA sequencing analyses.

DEGs were identified by the function “Find All Markers” or “Find Markers” in Seurat packages using “Wilcox” test methods and the Bonferroni correction. Biomarkers of different clusters were selected from DEGs with the adjusted *p*-value “p_val_adj ≤ 0.05” and log-transformed fold change “avg_logFC ≥ 1.0” for further analysis and visualization.

The transcription factors were predicted within 2000 bp upstream and 500 bp downstream of the transcription start sites for the DEGs using the JASPAR database [42] and TFBSTools [43]. The DEGs and transcription factors network were visualized using Cytoscape software (Cytoscape Consortium, San Diego, CA, USA; version 3.9.1).

### 4.6. Viral Gene Delivery

For rescue of SM22α expression, the Adeno-associated virus (AAV) serotype 2/9 containing a cDNA sequence specific for mouse Tagln (pcAAV-CMV-EGFP-P2A-Tagln-3 × FLAG-WPRE, AAV-SM22α) or negative control (pcAAV-CMV-EGFP-P2A-MCS-3 × FLAG-WPRE, AAV-Flag) were constructed and purchased from Obio Technology Company (Obio, Shanghai, China). The recombinant AAV constructs were injected into the *Sm22α*^−/−^ mice (10 weeks) via the tail vein at 5 × 10^9^ pfu/kg.

### 4.7. Behavioral Assays

All mice used for the tests were first evaluated for general health, including body weight and fur appearance. The mice were allowed to habituate to the testing room for 30 min before the commencement of every test.

#### 4.7.1. Open Field Test

The open field test was measured as described previously [44]. The mice were placed in an arena (50 cm × 50 cm × 50 cm) and the center (25 cm × 25 cm) region was artificially defined as the central region. The movement was monitored and recorded for 5 min using overhead ANY-maze video-tracking software (Stoelting Co, Wood Dale, IL, USA; version 4.5). Locomotor activity was evaluated as the total distance traveled. Anxiety-like behavior was defined by the distance traveled in the center compared to the distance traveled in the perimeter of the arena. The more time spent in the center, the more relaxed the animal.

#### 4.7.2. Novel Object Recognition Test

The novel object recognition test was performed as previously described [45]. Briefly, two identical objects were located symmetrically and set a distance apart in an enclosed arena. The mice were placed in the arena for familiar training to explore the objects freely within 5 min. Twenty-four hours after the training, one of the two previously used objects (familiar object) was replaced with a novel object that was different from the familiar object in shape, texture, and appearance. The movement was monitored and recorded using ANY-maze video-tracking software (Stoelting Co, Wood Dale, IL, USA; version 4.5). The recognition memory ability of the mouse to discriminate between familiar and novel objects was quantified by using the exploration time. The more time spent exploring the novel object, the better short-term learning and memory ability.

#### 4.7.3. Morris Water Maze Test

The Morris water maze test was performed as previously described [46]. The training was performed in a round, opacified, water-filled tube (120 cm in diameter) in an environment rich with extra maze cues. The mice were placed in the water maze with their paws touching the wall from four different starting positions in water consistently maintained at 19–23 °C. The tube was drained and cleaned every day after testing. The platform (10 cm in diameter) was fixed in the same spatial location 1 cm below the water surface. During the training period, the mice were allowed to freely swim for 60 s to find the platform using extra maze cues. Each mouse had trained four trials per day at 15 min intervals, and the daily data presented are the average of the four trials. The mice that failed to find the platform were guided to it and allowed to stay for 30 s. After each trial, the mice were dried and kept in a dry plastic holding cage filled with paper towels to allow them to completely dry off. Then, 24 h after the last training trial, the platform was removed, and the mice were tested for memory retention in a probe test. The swimming activity of each mouse was monitored using a video camera mounted overhead and was automatically recorded via ANY-maze video-tracking software (Stoelting Co, Wood Dale, IL, USA; version 4.5). As an evaluation criterion, the shorter the escape latency, the better the learning and memory ability; meanwhile, the longer spent in the target quadrant, the better the spatial memory ability.

### 4.8. Tissue Staining

Following the behavioral tests, the mice were sacrificed after deep anesthesia with sodium pentobarbital (60 mg/kg, i.p.), and the brain tissues were collected on ice and fixed in 4% paraformaldehyde, embedded in paraffin, and sliced into 5-µm sections.

#### 4.8.1. Hematoxylin and Eosin (H&E) Staining

Paraffin sections were sequentially immersed in xylene and a graded alcohol series for deparaffinization and then stained with hematoxylin and eosin (Leica Biosystems, Nussloch, Germany). After neutral balsam mounting, the sections were observed under an optical microscope and then photographed. A mean value was determined for each animal from at least three sections.

#### 4.8.2. Nissl Staining

Nissl staining was used to identify Nissl bodies in the neuronal cytoplasm. Paraffin sections were sequentially immersed in xylene and a graded alcohol series for deparaffinization and then stained with Nissl staining solution (Solarbio, Beijing, China) according to the manufacturer’s instructions. The sections were washed twice with distilled water and then 70% ethanol. After neutral balsam mounting, the sections were observed under an optical microscope and then photographed. A mean value was determined for each animal from at least three sections.

#### 4.8.3. Thioflavin-S Staining

An improved thioflavin-S staining protocol [47] was used to ensure reduced photobleaching and tissue damage. Briefly, the sections underwent pretreatment with KMnO_4_ for 4 min followed by 1% sodium borohydride for quenching. Then, the sections were stained with 0.05% thioflavin-S (Sigma, Saint Louis, MO, USA) in 50% ethanol in the dark for 8 min. Subsequently, the sections were successively rinsed in 80% alcohol and distilled water and post-treated with a high concentration of phosphate buffer for 30 min at 4 °C to alleviate photobleaching. Amyloid deposition was evaluated under a fluorescent microscope.

### 4.9. Cell Culture and Treatment

VSMCs were isolated from the aortas of the mice and cultured in a low-glucose Dulbecco’s-modified Eagle’s medium (DMEM) (Invitrogen, Waltham, MA, USA) supplemented with 10% fetal bovine serum (FBS, Gibco, Waltham, MA, USA), 100 U/mL of penicillin, and 100 μg/mL of streptomycin. The VSMCs were maintained at 37 °C in a humidified atmosphere containing 5% CO_2_, and only 3–7 passages of VSMCs were used in the experiments, unless otherwise noted. Serum starvation was carried out by withdrawing the serum and incubating the cells in 0.5% FBS for 24 h before stimulation with PDGF-BB (20 ng/mL, Sigma, Saint Louis, MO, USA). Human embryonic kidney (HEK-293A) cells were purchased from ATCC (American Type Culture Collection, VA, USA) and maintained in high-glucose DMEM supplemented with 10% FBS, 100 U/mL of penicillin, and 100 of μg/mL streptomycin. HEK-293A cells were transfected with Sox10 expression plasmids (Flag-SOX10-WT or Flag-SOX10-S24A, General Biosystems, Morrisville, NC, USA) using HighGene transfection reagent (ABclonal, Woburn, MA, USA) for 24 h, then treated with or without TNF-α (10 ng/mL, PeproTech, Cranbury, NJ, USA), cycloheximide (CHX, 10 μg/mL, Sigma, Saint Louis, MO, USA), and MG132 (10 μmol/L, Sigma, Saint Louis, MO, USA).

### 4.10. Ex Vivo Experiments and Inflammatory Models

For the ex vivo experiments, healthy brains and aortas from the mice were collected and immediately incubated in normal media for 2 h, maintained at 37 °C in a humidified atmosphere containing 5% CO_2_. Then, the brains and aortas were treated with PDGF-BB (60 ng/mL, Sigma, Saint Louis, MO, USA) to mimic inflammatory conditions for 48 h [12]. The control groups were treated with DMSO at 0.01%.

### 4.11. Western Blotting

Lysates from cells or tissue samples were prepared with a lysis buffer (Beyotime, Shanghai, China) and the protein concentrations were determined with BCA protein assays. Equal amounts of protein (30–100 μg) were separated by 8%, 10%, or 12% SDS-PAGE and electro-transferred to a PVDF membrane (Merck Millipore, Darmstadt, Germany). After blocking with 5% milk in TBST, the membranes were incubated with primary antibodies at 4 °C overnight, and then with the HRP-conjugated secondary antibody for 1 h at room temperature. The blots were visualized using the Image Quant LAS 4000 detection system (GE Healthcare, Chicago, IL, USA). Band intensities were quantified with Image Pro Plus software (Media Cybernetics, Rockville, MD, USA; version 6.0). These experiments were replicated at least three times. The following primary antibodies were used: SM22α (Abcam, Cambridge, UK; 1:1000), OPN (Santa Cruz, CA, USA; 1:500), IL-6 (Proteintech, Wuhan, China; 1:500), Sox10 (Santa Cruz, CA, USA; 1:50), and Flag (MBL International, Woburn, MA, USA; 1:2000).

### 4.12. Statistical Analysis

All data are presented as the mean ± SD and were analyzed with GraphPad Prism software (GraphPad Software, San Diego, CA, USA; version 8.2.0). A two-tailed unpaired Student’s *t*-test was used to compare the two groups. A two-way ANOVA followed by Tukey’s post hoc test was used for multiple group comparisons. A value of *p* < 0.05 was considered statistically significant.

## 5. Conclusions

In summary, this study demonstrates that SM22α-related VSMC dysfunction is associated with neurological impairment and that the rescuing of SM22α expression ameliorates cognitive impairment in mice. Sox10 enhances the proinflammatory phenotypic switching of VSMCs with SM22α loss, contributing to exaggerated inflammation both in the aorta and brain. SM22α-Sox10 axis may be a novel regulator of neurocognitive impairment through modulating VSMC phenotypes and may be a potential control point in dementia.

## Data Availability

The datasets produced and/or analyzed during the current study are available from the corresponding author on reasonable request.

## Supplementary Materials

The following supporting information can be downloaded at: https://www.mdpi.com/article/10.3390/ijms24087117/s1.

## Abbreviations

AAV	Adeno-associated virus
AD	Alzheimer’s disease
Aβ	Amyloid-β
BP	Biological processes
CC	Cellular components
CE	Cholesterol esters
CHX	Cycloheximide
DEGs	Differentially expressed genes
DHA	Docosahexaenoic acid
GO	Gene ontology
HMG	High-mobility group
IL-6	Interleukin-6
KEGG	Kyoto Encyclopedia of Genes and Genomes
MF	Molecular functions
MWM	Morris water maze test
NOR	Novel object recognition test
NVUs	Neurovascular units
OFT	Open field test
PC	Phosphatidylcholine
PDGF-BB	Platelet-derived growth factor-BB
PE	Phosphatidylethanolamine
SM22α	Smooth muscle 22 alpha
*Sm22α* ^−/−^	SM22α knockout
Sox10	SRY-related HMG-box gene 10
VSMCs	Vascular smooth muscle cells
WT	Wild-type

## References

[1] Cheng Y.W., Chen T.F., Chiu M.J. (2017). From mild cognitive impairment to subjective cognitive decline: Conceptual and methodological evolution. Neuropsychiatr. Dis. Treat..

[2] Livingston G., Huntley J., Sommerlad A., Ames D., Ballard C., Banerjee S., Brayne C., Burns A., Cohen-Mansfield J., Cooper C. (2020). Dementia prevention, intervention, and care: 2020 report of the Lancet Commission. Lancet.

[3] Tian Z., Ji X., Liu J. (2022). Neuroinflammation in Vascular Cognitive Impairment and Dementia: Current Evidence, Advances, and Prospects. Int. J. Mol. Sci..

[4] van der Flier W.M., Skoog I., Schneider J.A., Pantoni L., Mok V., Chen C.L.H., Scheltens P. (2018). Vascular cognitive impairment. Nat. Rev. Dis. Prim..

[5] Azarpazhooh M.R., Avan A., Cipriano L.E., Munoz D.G., Sposato L.A., Hachinski V. (2018). Concomitant vascular and neurodegenerative pathologies double the risk of dementia. Alzheimers Dement..

[6] Strandberg T.E., Tienari P.J., Kivimäki M. (2020). Vascular and Alzheimer Disease in Dementia. Ann. Neurol..

[7] Frismantiene A., Philippova M., Erne P., Resink T.J. (2018). Smooth muscle cell-driven vascular diseases and molecular mechanisms of VSMC plasticity. Cell. Signal..

[8] Wirka R.C., Wagh D., Paik D.T., Pjanic M., Nguyen T., Miller C.L., Kundu R., Nagao M., Coller J., Koyano T.K. (2019). Atheroprotective roles of smooth muscle cell phenotypic modulation and the TCF21 disease gene as revealed by single-cell analysis. Nat. Med..

[9] Zhang J.C., Kim S., Helmke B.P., Yu W.W., Du K.L., Lu M.M., Strobeck M., Yu Q., Parmacek M.S. (2001). Analysis of SM22alpha-deficient mice reveals unanticipated insights into smooth muscle cell differentiation and function. Mol. Cell. Biol..

[10] Fu Y., Liu H.W., Forsythe S.M., Kogut P., McConville J.F., Halayko A.J., Camoretti-Mercado B., Solway J. (2000). Mutagenesis analysis of human SM22: Characterization of actin binding. J. Appl. Physiol..

[11] Han M., Dong L.H., Zheng B., Shi J.H., Wen J.K., Cheng Y. (2009). Smooth muscle 22 alpha maintains the differentiated phenotype of vascular smooth muscle cells by inducing filamentous actin bundling. Life Sci..

[12] Aguilar-Pineda J.A., Vera-Lopez K.J., Shrivastava P., Chávez-Fumagalli M.A., Nieto-Montesinos R., Alvarez-Fernandez K.L., Goyzueta Mamani L.D., Davila Del-Carpio G., Gomez-Valdez B., Miller C.L. (2021). Vascular smooth muscle cell dysfunction contribute to neuroinflammation and Tau hyperphosphorylation in Alzheimer disease. iScience.

[13] Kanehisa M., Goto S., Sato Y., Furumichi M., Tanabe M. (2012). KEGG for integration and interpretation of large-scale molecular data sets. Nucleic Acids Res..

[14] Naudí A., Cabré R., Jové M., Ayala V., Gonzalo H., Portero-Otín M., Ferrer I., Pamplona R. (2015). Lipidomics of human brain aging and Alzheimer’s disease pathology. Int. Rev. Neurobiol..

[15] Zhang D.D., Song Y., Kong P., Xu X., Gao Y.K., Dou Y.Q., Weng L., Wang X.W., Lin Y.L., Zhang F. (2021). Smooth muscle 22 alpha protein inhibits VSMC foam cell formation by supporting normal LXRα signaling, ameliorating atherosclerosis. Cell Death Dis..

[16] Fang L., Li Y., Ma L., Xu Q., Tan F., Chen G. (2021). GRNdb: Decoding the gene regulatory networks in diverse human and mouse conditions. Nucleic Acids Res..

[17] Cronin J.C., Loftus S.K., Baxter L.L., Swatkoski S., Gucek M., Pavan W.J. (2018). Identification and functional analysis of SOX10 phosphorylation sites in melanoma. PLoS ONE.

[18] Zlokovic B.V. (2011). Neurovascular pathways to neurodegeneration in Alzheimer’s disease and other disorders. Nat. Rev. Neurosci..

[19] Owens G.K. (1995). Regulation of differentiation of vascular smooth muscle cells. Physiol. Rev..

[20] Wang G., Jacquet L., Karamariti E., Xu Q. (2015). Origin and differentiation of vascular smooth muscle cells. J. Physiol..

[21] Shen J., Yang M., Ju D., Jiang H., Zheng J.P., Xu Z., Li L. (2010). Disruption of SM22 promotes inflammation after artery injury via nuclear factor kappaB activation. Circ. Res..

[22] Shu Y.N., Zhang F., Bi W., Dong L.H., Zhang D.D., Chen R., Lv P., Xie X.L., Lin Y.L., Xue Z.Y. (2015). SM22α inhibits vascular inflammation via stabilization of IκBα in vascular smooth muscle cells. J. Mol. Cell. Cardiol..

[23] Shu Y.N., Dong L.H., Li H., Pei Q.Q., Miao S.B., Zhang F., Zhang D.D., Chen R., Yin Y.J., Lin Y.L. (2017). CKII-SIRT1-SM22α loop evokes a self-limited inflammatory response in vascular smooth muscle cells. Cardiovasc. Res..

[24] Dong L.H., Wen J.K., Liu G., McNutt M.A., Miao S.B., Gao R., Zheng B., Zhang H., Han M. (2010). Blockade of the Ras-extracellular signal-regulated kinase 1/2 pathway is involved in smooth muscle 22 alpha-mediated suppression of vascular smooth muscle cell proliferation and neointima hyperplasia. Arterioscler. Thromb. Vasc. Biol..

[25] Zhong L., He X., Si X., Wang H., Li B., Hu Y., Li M., Chen X., Liao W., Liao Y. (2019). SM22α (Smooth Muscle 22α) Prevents Aortic Aneurysm Formation by Inhibiting Smooth Muscle Cell Phenotypic Switching through Suppressing Reactive Oxygen Species/NF-κB (Nuclear Factor-κB). Arterioscler. Thromb. Vasc. Biol..

[26] Lv P., Miao S.B., Shu Y.N., Dong L.H., Liu G., Xie X.L., Gao M., Wang Y.C., Yin Y.J., Wang X.J. (2012). Phosphorylation of smooth muscle 22α facilitates angiotensin II-induced ROS production via activation of the PKCδ-P47phox axis through release of PKCδ and actin dynamics and is associated with hypertrophy and hyperplasia of vascular smooth muscle cells in vitro and in vivo. Circ. Res..

[27] Chen R., Zhang F., Song L., Shu Y., Lin Y., Dong L., Nie X., Zhang D., Chen P., Han M. (2014). Transcriptome profiling reveals that the SM22α-regulated molecular pathways contribute to vascular pathology. J. Mol. Cell. Cardiol..

[28] Haseeb A., Lefebvre V. (2019). The SOXE transcription factors-SOX8, SOX9 and SOX10-share a bi-partite transactivation mechanism. Nucleic Acids Res..

[29] Weider M., Wegner M. (2017). SoxE factors: Transcriptional regulators of neural differentiation and nervous system development. Semin. Cell Dev. Biol..

[30] Pingault V., Zerad L., Bertani-Torres W., Bondurand N. (2022). SOX10: 20 years of phenotypic plurality and current understanding of its developmental function. J. Med. Genet..

[31] Shakhova O., Zingg D., Schaefer S.M., Hari L., Civenni G., Blunschi J., Claudinot S., Okoniewski M., Beermann F., Mihic-Probst D. (2012). Sox10 promotes the formation and maintenance of giant congenital naevi and melanoma. Nat. Cell Biol..

[32] Cronin J.C., Watkins-Chow D.E., Incao A., Hasskamp J.H., Schönewolf N., Aoude L.G., Hayward N.K., Bastian B.C., Dummer R., Loftus S.K. (2013). SOX10 ablation arrests cell cycle, induces senescence, and suppresses melanomagenesis. Cancer Res..

[33] Graf S.A., Busch C., Bosserhoff A.K., Besch R., Berking C. (2014). SOX10 promotes melanoma cell invasion by regulating melanoma inhibitory activity. J. Investig. Dermatol..

[34] Wang D., Wu F., Yuan H., Wang A., Kang G.J., Truong T., Chen L., McCallion A.S., Gong X., Li S. (2017). Sox10(+) Cells Contribute to Vascular Development in Multiple Organs-Brief Report. Arterioscler. Thromb. Vasc. Biol..

[35] Tang Z., Wang A., Yuan F., Yan Z., Liu B., Chu J.S., Helms J.A., Li S. (2012). Differentiation of multipotent vascular stem cells contributes to vascular diseases. Nat. Commun..

[36] Erkinjuntti T., Gauthier S. (2009). The concept of vascular cognitive impairment. Front. Neurol. Neurosci..

[37] Zhang J., Zhong W., Cui T., Yang M., Hu X., Xu K., Xie C., Xue C., Gibbons G.H., Liu C. (2006). Generation of an adult smooth muscle cell-targeted Cre recombinase mouse model. Arterioscler. Thromb. Vasc. Biol..

[38] Wiśniewski J.R., Zougman A., Nagaraj N., Mann M. (2009). Universal sample preparation method for proteome analysis. Nat. Methods.

[39] Bligh E.G., Dyer W.J. (1959). A rapid method of total lipid extraction and purification. Can. J. Biochem. Physiol..

[40] Song J.W., Lam S.M., Fan X., Cao W.J., Wang S.Y., Tian H., Chua G.H., Zhang C., Meng F.P., Xu Z. (2020). Omics-Driven Systems Interrogation of Metabolic Dysregulation in COVID-19 Pathogenesis. Cell Metab..

[41] Gao Y.K., Guo R.J., Xu X., Huang X.F., Song Y., Zhang D.D., Chen N., Wang X.W., Liang C.X., Kong P. (2022). A regulator of G protein signaling 5 marked subpopulation of vascular smooth muscle cells is lost during vascular disease. PLoS ONE.

[42] Sandelin A., Alkema W., Engström P., Wasserman W.W., Lenhard B. (2004). JASPAR: An open-access database for eukaryotic transcription factor binding profiles. Nucleic Acids Res..

[43] Tan G., Lenhard B. (2016). TFBSTools: An R/bioconductor package for transcription factor binding site analysis. Bioinformatics.

[44] Zhang B., Wang L., Zhan A., Wang M., Tian L., Guo W., Pan Y. (2021). Long-term exposure to a hypomagnetic field attenuates adult hippocampal neurogenesis and cognition. Nat. Commun..

[45] Jessberger S., Clark R.E., Broadbent N.J., Clemenson G.D., Consiglio A., Lie D.C., Squire L.R., Gage F.H. (2009). Dentate gyrus-specific knockdown of adult neurogenesis impairs spatial and object recognition memory in adult rats. Learn Mem..

[46] Lee S.E., Simons S.B., Heldt S.A., Zhao M., Schroeder J.P., Vellano C.P., Cowan D.P., Ramineni S., Yates C.K., Feng Y. (2010). RGS14 is a natural suppressor of both synaptic plasticity in CA2 neurons and hippocampal-based learning and memory. Proc. Natl. Acad. Sci. USA.

[47] Sun A., Nguyen X.V., Bing G. (2002). Comparative analysis of an improved thioflavin-s stain, Gallyas silver stain, and immunohistochemistry for neurofibrillary tangle demonstration on the same sections. J. Histochem. Cytochem..

