# Supplementary figures and images for: SM22α Deletion Contributes to Neurocognitive Impairment in Mice through Modulating Vascular Smooth Muscle Cell Phenotypes
Source: Int J Mol Sci. 2023 Apr 12;24(8):7117. doi: 10.3390/ijms24087117 (PMC10138350; doi:10.3390/ijms24087117)

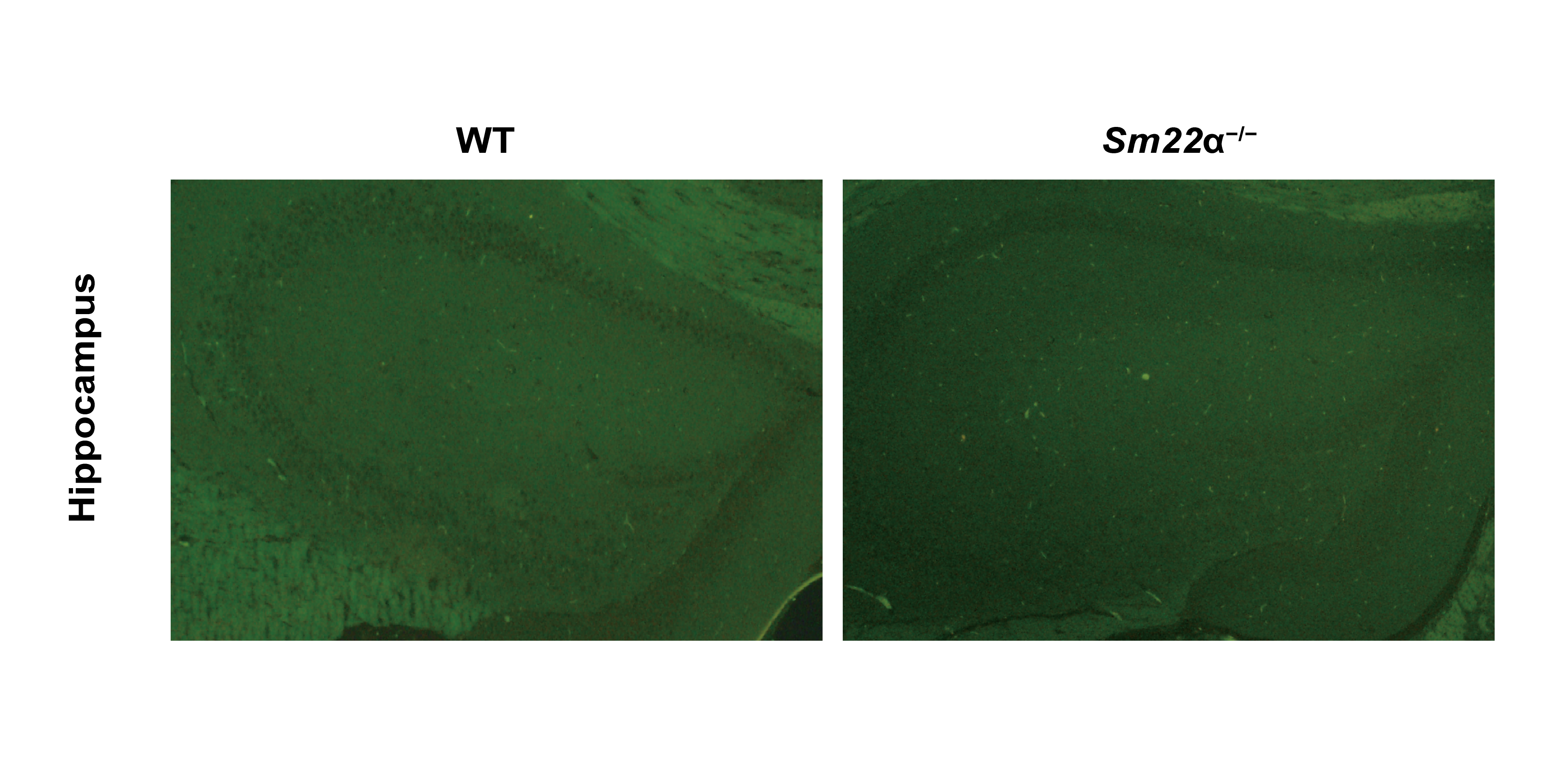

Supplement: Supplementary file 1 [file ijms-24-07117-s001.zip › ijms-2310368-Figure S1.tif]
